# Toward Interoperable Digital Medication Records on Fast Healthcare Interoperability Resources: Development and Technical Validation of a Minimal Core Dataset

**DOI:** 10.2196/64099

**Published:** 2025-05-09

**Authors:** Eduardo Salgado-Baez, Raphael Heidepriem, Renate Delucchi Danhier, Eugenia Rinaldi, Vishnu Ravi, Akira-Sebastian Poncette, Iris Dahlhaus, Daniel Fürstenau, Felix Balzer, Sylvia Thun, Julian Sass

**Affiliations:** 1 Department of Anesthesiology and Intensive Care Medicine (CVK/CCM) Charité - Universitätsmedizin Berlin Berlin Germany; 2 Core Unit Digital Medicine and Interoperability Berlin Institute of Health Charité - Universitätsmedizin Berlin Berlin Germany; 3 Institute for Diversity Studies TU Dortmund University Dortmund Germany; 4 Stanford Mussallem Center for Biodesign Stanford University Stanford United States; 5 Institute of Medical Informatics Charité - Universitätsmedizin Berlin Berlin Germany; 6 School of Business & Economics Freie Universität Berlin Berlin Germany

**Keywords:** FHIR, FAIR, standardization, dataset, electronic health records, digital, medication records, technical, validation, medication error, Fast Healthcare Interoperability Resources, Findability, Accessibility, Interoperability, and Reusability, software

## Abstract

**Background:**

Medication errors represent a widespread, hazardous, and costly challenge in health care settings. The lack of interoperable medication data within and across hospitals not only creates an administrative burden through redundant data entry but also increases the risk of errors due to human mistakes, imprecise data transformations, and misinterpretations. While digital solutions exist, fragmented systems and nonstandardized data hinder effective medication management.

**Objective:**

This study aimed to assess medication data available across the multiple systems of a large university hospital, identify a minimum dataset with the most relevant information, and propose a standard interoperable FHIR-based solution that can import and transfer information from a standardized drug master database to various target systems.

**Methods:**

Medication data from all relevant departments of a large German hospital were thoroughly analyzed. To ensure interoperability, data elements for developing a minimum dataset were defined based on relevant medication identifiers, the Health Level 7 Fast Health Interoperability Resources (HL7 FHIR) standard, and the German Medical Informatics Initiative (MII) specifications. To enhance medication identification accuracy, the dataset was further enriched with information from Germany’s most comprehensive drug database and European Standard Drug Terms (EDQM) to further enrich medication identification accuracy. Finally, data on 60 frequently used medications in the institution were systematically extracted from multiple medication systems used in the institution and integrated into a new structured, dedicated database.

**Results:**

The analysis of all the available medication datasets within the institution identified 7964 drugs. However, limited interoperability was observed due to a fragmented local IT infrastructure and challenges in medication data standardization. Data integrated and available in the new structured medication dataset with key elements to ensure data identification accuracy and interoperability, successfully enabled the generation of medication order messages, ensuring medication interoperability, and standardized data exchange.

**Conclusions:**

Our approach addresses the lack of interoperability in medication data and the need for standardized data exchange. We propose a minimum set of data elements aligned with German and international coding systems to be used in combination with the FHIR standard for processes such as the digital transfer of discharge medication prescriptions from intensive care units to general wards, which can help to reduce medication errors and enhance patient safety.

## Introduction

### Medication Errors

In 2022, approximately seventeen million patients were treated in German hospitals, of which more than 2 million were admitted to an intensive care unit (ICU) for the treatment of life-threatening conditions due to the dysfunction of one or more organs [1], which inevitably requires the administration of multiple medicaments [2]. As a result of the complexity of the medication process, medication errors have been a frequent and safety-relevant problem in medical care [3]. The first major groundbreaking study that quantified this situation was the “To Err is Human: Building a Safer Health Care System” report conducted by the Institute of Medicine in the United States [4]. In recent years, as reported by the Joint Commission in 2020 [5], medication errors are involved in 5.4% of all severe injuries or patient deaths, with a total annual cost of USD 42 billion according to estimates of the World Health Organization (WHO) [6,7].

This situation is caused, among other factors, by a lack of interoperable pharmaceutical documentation systems [8] as well as because many steps of the process (ie, prescription, transmission of the information, and documentation) are still highly dependent on manual input and prevent the implementation of medication reconciliation systems (MedRec). As a result, medication errors and adverse drug events related to unintended medication discrepancies in electronic health records (EHR) are still a major public health problem and are listed unsurprisingly among the top 10 health technology hazards for 2020 by the Emergency Care Research Institute [9-11].

### Interoperability, Standardization, and Data Access

There is evidence that interoperable IT systems and the use of international standards can help to mitigate medication errors and facilitate the exchange of information across different platforms and software used for drug prescription and administration [12].

The Healthcare Information and Management Systems Society defines interoperability as the capacity of at least 2 IT systems to communicate by exchanging standardized data, allowing the use of the exchanged information [13]. Its implementation in the medication field is critical for the digital transformation of drug prescription, administration, and research [14], and relies on the employment of internationally standardized data and terminologies.

For the health care industry, Health Level 7 (HL7) has provided a comprehensive set of international standards for clinical and administrative data transfer across software that numerous health care providers employ to promote data exchange and interoperability, including our hospital and its EHR. It remains the most popular and widespread health care framework, improving diagnostics, therapy, and patient safety and outcomes. [15,16].

In 2011, due to the rapidly growing amount of health data, HL7 started developing the Fast Interoperability Resources (FHIR), a standard that addresses the need for faster and better methods for interoperable data exchange.

FHIR was designed to be flexible and adaptable, making this standard easy to implement and suitable for a wide range of clinical processes. It uses a modern web-based application programming interface (API) [17].

For global standardization and interoperability of medication data, the International Organization for Standardization (ISO) has been developing the Identification of Medicinal Products (IDMP) since 2012, providing a suite of 5 standards (ISO 11615, ISO 11616, ISO 11238, ISO 11239, and ISO 11240) for identifying and exchanging the information of each medicinal product for human use throughout the world [18-23].

Although these standards were created for pharmacovigilance purposes, over the years, the ISO IDMP standards have come to support various other activities, including those related to the norms for the unique identification and exchange of regulated information about medicinal and pharmaceutical products.

In Europe, the European Medicines Agency (EMA) is the institution responsible for gradually implementing ISO IDMP standards through a plan and services based on four domains of master data in pharmaceutical regulatory processes (Substance, Product, Organization, and Referential [SPOR]), which are crucial to ensuring interoperability [24,25].

In cooperation with the EMA, the European Directorate for the Quality of Medicines and HealthCare (EDQM) promotes the development of a common pharmacopoeia in Europe. In this context and as part of the implementation of quality standards for medicines, the EDQM [26] released the EDQM standard terms, comprising terms and definitions to describe (among others) pharmaceutical dose forms, routes and methods of administration, administration devices, and units of presentation [27], providing a legal and scientific basis for quality control of medications from development to marketing as well as a framework for the safe use of medicines on patients [28].

According to the EMA, the standards and services mentioned above enable operational benefits, especially considering their positive impact on the regulation of medicines and the simplification of medication information exchange in Europe and internationally. This higher degree of standardization and regulation aims to provide a framework to improve data transfer, patient safety, and public health [29].

**Figure 1:**
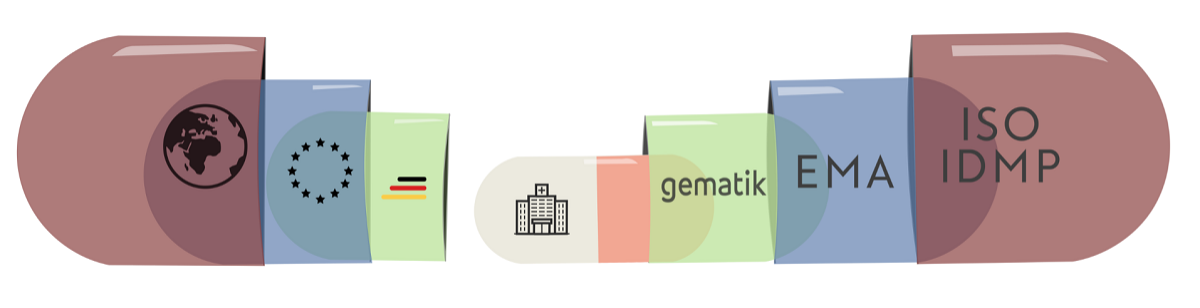
Visualization of the standardization levels of medicinal products. EDQM: European Directorate for the Quality of Medicines; EMA: European Medicines Agency; IDMP: Identification of Medicinal Products; ISO: International Standardization Organization.

At the national level, the German Medical Informatics Initiative (MII) is a federal effort dedicated to digitizing the German health care system. As a part of this initiative, a medication task force group has been established to leverage standardized frameworks for different medication processes [30]. In addition, the gematik [31] is the current organization responsible for the federal telematics infrastructure (TI), a central platform for digital applications. One central function of the gematik is the definition and enforcement by law of binding standards for services, components, and applications for the TI and the implementation of standards’ specifications like FHIR, SNOMED CT (Systematized Nomenclature of Medicine Clinical Terms), and LOINC (Logical Observation Identifiers Names and Codes). In the case of medication, the gematik is developing the ISiK-Modules (Informationstechnische Systeme in Krankenhäusern; English: Information technology systems in hospitals) based on FHIR resources for exchanging medication data [32]. An overview of the different standardization levels is presented in Figure 1.

Responding to the need to make digital data assets and their associated metadata more usable by machines and reusable by humans, Wilkinson et al [33], developed a set of 15 guiding principles for scientific data management and stewardship, which are grouped into the four higher principles of Findability, Accessibility, Interoperability, and Reusability (FAIR). Implementing the FAIR Principles is relevant to improving efficiency and access to research and health care data. Since their publication, many authors have highlighted the benefits of its adoption [34-36].

In contrast, the most comprehensive medication records in Germany, such as the ABDA (Federal Union of German Associations of Pharmacists) or ABDA-MED (ABDA Medication Database) and MMI Pharmaindex Plus, are commercial, proprietary databases that can only be accessed under license, limiting public availability, and requiring authorized usage for comprehensive pharmaceutical data retrieval [37,38].

### Objective

As our institution is not immune to the risk of medication errors, and modern IT solutions for medication management are set to be gradually implemented in the coming years, this paper aims to (1) assess the extent to which medication systems in our hospital use standardized medication data and comply with established standards and (2) propose a proof-of-concept FHIR-based interoperable solution with noncommercial, standardized, minimal, and FAIR medication data to enhance medication interoperability.

## Methods

### Departments Involved

The key units involved in the medication process were identified by the Chief Medical Informatics Officer (CMIO) team before the start of the study. Therefore, no additional partners were required for this project. The study included the IT department, the pharmacy, the PDMS used throughout the institution’s ICUs, and selected general wards. It examined their databases, focusing on data quality, standardization, storage, governance, and both semantic and technical interoperability. The medication data provided and extracted from these datasets were systematically structured and classified to develop a standardized database.

### Selection of Drugs and Data Components for the Standardized Database

As medication datasets are not freely accessible by default for safety and commercial reasons, governance staff responsible for managing medication records on a particular stakeholder’s software provided access to the data. They provided the authors with access to the medication databases, detailed information about the software communication protocols for exchanging data, and the methods used to keep the information up to date. The selection criteria for drugs included in this study’s novel and standardized database were based on the 60 most frequently administered medications in an anesthesiology ICU. These drugs are documented using quick-access buttons in the medication section of the ICU Patient Data Management System (PDMS), designed for recording commonly used medications. For this study, data related to these drugs were extracted from the datasets of the units participating in the study, which used different systems, and were integrated into our database.

We searched and selected data components from multiple datasets used in the institution to develop a minimal, FAIR, and standardized dataset capable of accurately identifying every single drug with the least possible amount of data. Our selection was based on the Medication and Medication Request resources of the HL7 FHIR standard, given a future modernization of medication software. The following data elements for each drug were included: drug name, institution-specific product number, Pharmacy Central Number (PZN), Pharmacy Product Number (PPN), Anatomical Therapeutic Chemical (ATC), and the German Drug Substance Catalog (ASK) code.

Data provided from institutional databases was complemented with records drawn from the most comprehensive German medication register, the ABDA or ABDA-MED proprietary database [39]. To ensure compatibility with European standards [40], we added information available on the EDQM standard terms [41], incorporating standardized data on administration methods, intended site, and routes of administration. Ultimately, all data was compiled into a single medication core dataset [42], used to generate FHIR order message prototypes.

**Figure 2:**
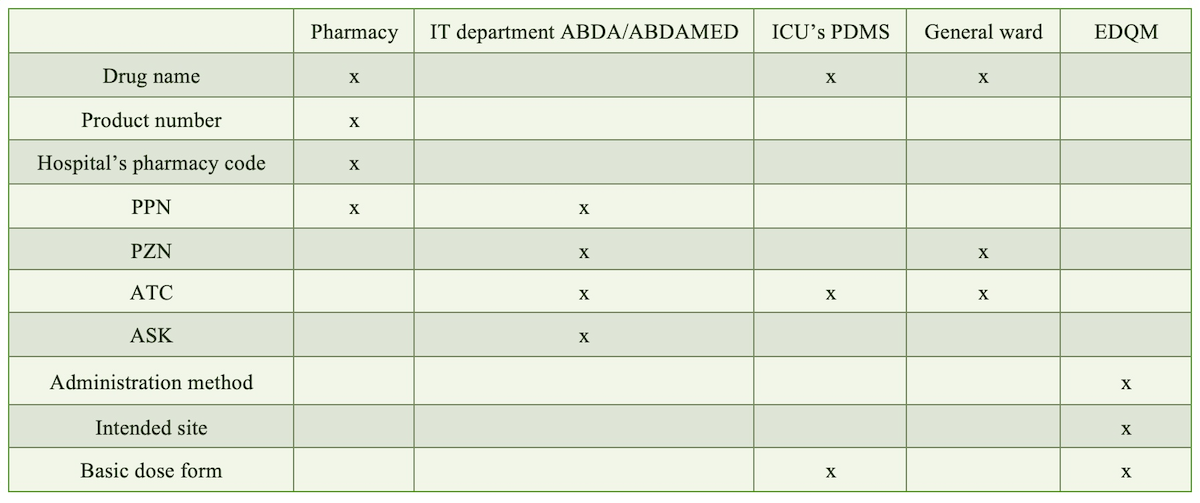
Source of specific medication data and included data elements in the standardized database. ABDA: Federal Union of German Associations of Pharmacists; ABDA-MED: ABDA Medication Database; ASK: German drug substance catalog; ATC: Anatomical Therapeutic Chemical; EDQM: European Directorate for the Quality of Medicines and HealthCare; PPN: Pharmacy Product Number; PZN: Pharmacy Central Number.

As summarized in Figure 2, the different pieces of information were gathered according to their availability to the different stakeholders as well as national and international databases.

### Ethical Considerations

This work was conceived as part of an operative medication project at Charité - Universitätsmedizin Berlin and the Berlin Institute of Health (BIH) to assess the status quo and modernize medication software and management across the institution. The study did not gather patient data and focused solely on general medication records that included drug information for the analysis. Consequently, the authors did not pursue approval from the institutional ethics committee.

## Results

### Overview

Multiple medication software and datasets were used throughout the hospital. Medication software was provided by different companies with limited data compatibility and transfer capabilities between them. Different departments maintained medication records separately. For these reasons, it was necessary to contact the hospital staff responsible for each medication software and dataset used to analyze medication.

The EHR maintained the relevant medication master data by using the commercial medication database ABDA or ABDA-MED as the basis for the internal medication data managed by the IT department. A critical unit relying on this information was the pharmacy. The pharmacy information system (PIS) could exchange data directly with the ABDA-MED database, demonstrating interoperability through the shared use of the same database.

However, we found that the PIS lacked an interface to share data directly with other systems, such as the ICU PDMS or medication software in the general wards. Its working database was a spreadsheet listing 3146 medications available in the hospital’s drug storage, containing four data attributes: institution-specific material number, PZN, drug name, and manufacturer. In other words, apart from communicating with the main database, the pharmacy was isolated and unable to exchange information in an interoperable manner with any other system involved in the medication process.

The assessment of the study ICU showed that medications were always prescribed and documented in digital form using the ICU’s PDMS. Nonetheless, the digital user interface (UI) was founded on a spreadsheet-based database containing information about 4818 drugs, which was compiled and imported manually by a senior physician and an employee from the division of clinical procedures.

The PDMS database contained nonstandardized information, including drug ID, PDMS name, trade name, generic name, active ingredient, medication family, main administration method, application form, calories, weight, dosage, and incomplete administrative data. The PDMS was also isolated and noninteroperable, as it lacked an IT interface to transfer data to the EHR, PIS, or general wards.

The medication workflow in the examined general wards required a medication application to digitalize the medications prescribed to a particular patient. This system used data stored in another proprietary dataset, the MMI Pharmaindex, comprising the following information: ATC and *ICD-10* (*International Statistical Classification of Diseases, Tenth Revision*) codes, company type, country of production, molecule type, pharmaceutical form, molecule unit, molecule nature, and package unit. This software was incompatible with the other medication applications mentioned earlier due to the absence of an interface, and its data access was highly restricted.

**Figure 3:**
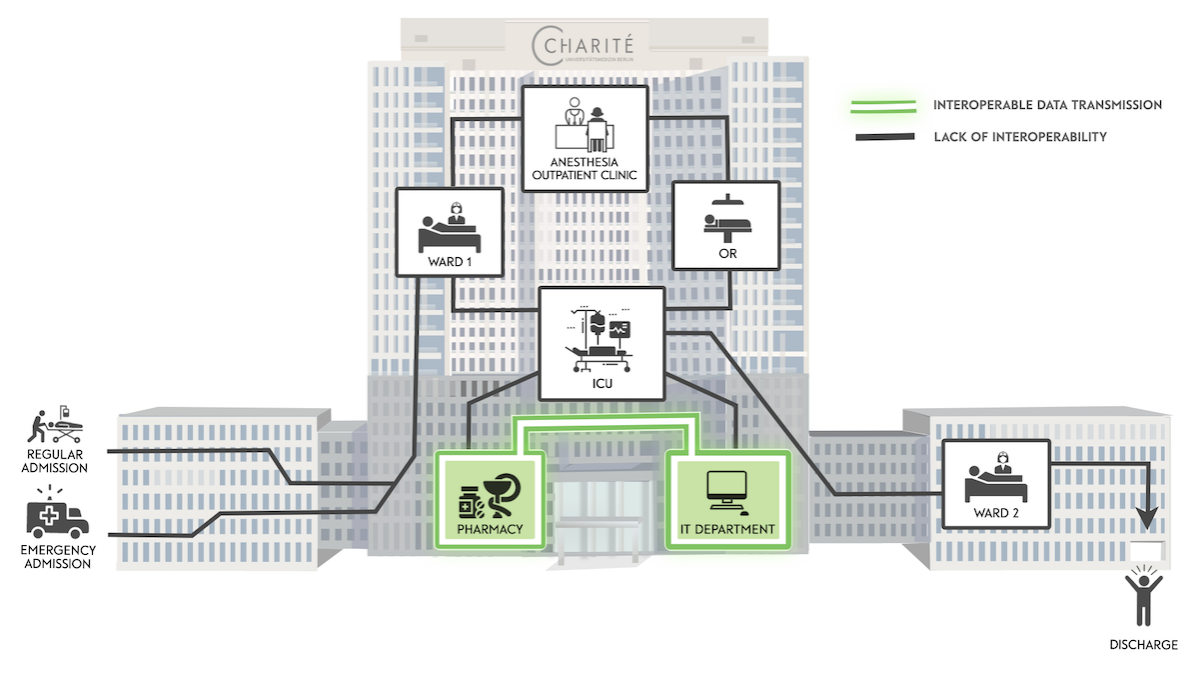
Visualization of pharmaceutical data flow within the assessed organization: Green lines represent departments where interoperable data transmission has been successfully implemented, while black lines highlight areas where interoperability is still lacking. The visualization emphasizes the significant work that remains to be done.

In summary, we examined communication flow concerning medication in a large hospital. This process spans across several phases and departments, such as admission, ward, anesthesia, ICU, pharmacy, inpatient stay, and discharge as shown in Figure 3. Little interoperability was found across the examined systems; medication information was isolated and needed human intervention to be exchanged. The absence of a central database with uniform medication standards led to the maintenance of a wide range of isolated, noninteroperable datasets by different stakeholders, except for PIS and the IT Department, which used the ABDA-MED database.

**Figure 4:**
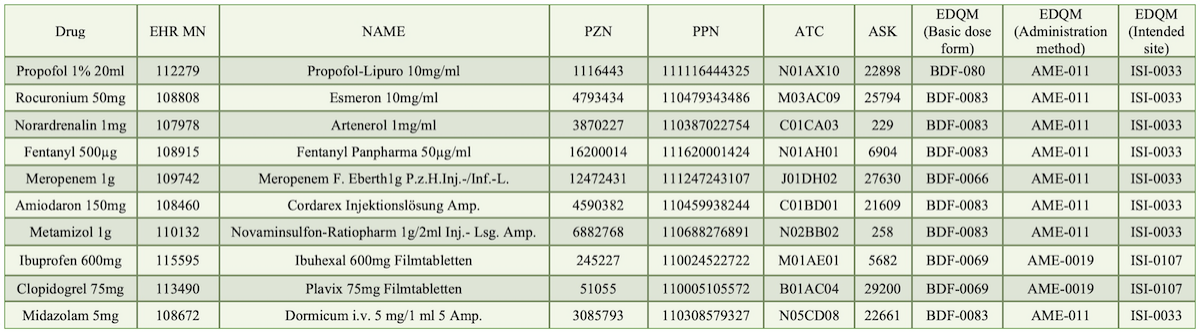
Extract of 10 drugs and their standardized identification codes. ASK: German drug substance catalog; ATC: Anatomical Therapeutic Chemical; EDQM: European Directorate for the Quality of Medicines and HealthCare; MN: Material Number; PPN: Pharmacy Product Number; PZN: Pharmacy Central Number.

After analyzing various databases and identifying a minimum dataset of the most relevant medication-related information, we developed a standardized medication database. This novel database incorporates a minimal set of FAIR-compliant data for 60 drugs frequently used in our hospital’s ICUs, enabling accurate identification of each drug. The final database includes the following data: drug name, institution-specific product number, PZN, PPN, ATC, ASK, and EDQM codes for administration method, intended site of use, and routes of administration. Examples of 10 drugs are shown in Figure 4. A full version of the database is accessible on Figshare [43].

### Technical Validation and Proof-of-Concept Development

By using the developed database together with HL7 FHIR specifications, we generated order messages for communication within medication software (Multimedia Appendices 1 and 2). These messages incorporated drug-specific, standardized data essential for the exchange of information between various software and applications. These are the messages that each source node in the network would generate to be validated and forwarded to its destination by the FHIR server.

Another essential aspect identified during the analysis of medication prescriptions in the ICU was the standardization of custom formulations, where a composition of 2 or more drugs must be represented. These are frequently used in an ICU setting and thus have high therapeutic relevance. In this regard, an agreement between the IT and pharmacy departments was established, introducing a new material number for identifying compounded medications to comply with the proposed standardization (Multimedia Appendices 3 and 4).

## Discussion

### Principal Results

This study assessed pharmaceutical records in a large university hospital and proposes a standardized solution for digitalization and interoperability across various medication systems and datasets. Leveraging the HL7 FHIR standard, it outlines the development of a proof-of-concept solution to enhance seamless data exchange and integration.

As described by Lehne et al [44], noninteroperable systems lead to undesired outcomes, such as redundant data storage, manual data maintenance, data discrepancies, difficulty in drug tracking, billing problems, inefficiency, and resource waste due to the high costs and time demands on trained personnel. Manual entry and transmission of medication information can contribute to clinical staff dissatisfaction and pose risks of patient harm, including errors that could result in injuries or death [45]. In addition, these practices hinder extensive medication research and artificial intelligence (AI) adoption [46-48], making large-scale studies nearly unfeasible. They also impede the implementation of closed-loop medication administration (CLMA) and MedRec systems, which could significantly reduce medication administration errors [49-51].

Integrating documentation software across different clinical departments (eg, emergency room, normal ward, operating room, and ICU) and including nonclinical areas (eg, pharmacy or IT department) in data management remains an open challenge. Therefore, a standardized central database and an interoperable interface enabling seamless communication among various software systems in hospital settings are needed.

At the time of analysis, our clinic lacked a central medication database that considered all stakeholders in the prescription process or the scientific reuse of data across departments, leading to data isolation and lack of interoperability. This issue is a crucial obstacle to enhancing patient safety and quality of care. Consequently, the institution is undergoing a significant overhaul of medication processes with FHIR standards playing a significant role.

These challenges are not unique to our institution but represent a global issue stemming from the general lack of standardization [52]. Efforts such as ISO IDMP standards, EMA medication guidelines, and local initiatives like MII and gematik [53] in Germany are crucial and should be considered when developing any IT medication solution. Overcoming obstacles such as the absence of public medication databases is a significant public health concern, as it directly affects data accessibility, exchange, transparency, management [54], and reuse.

To tackle these issues, we propose a FHIR R4–based solution designed to seamlessly import and transfer information from a standardized drug master database to various target systems in a configurable manner. Developed with an international perspective, this approach aims to enhance interoperability and standardization in medication data exchange.

The FHIR profiles used derive from the German MII, which is currently only used for secondary purposes by our large hospital. We rely on MII assets for terminology services, validation, and profile conformance.

To implement this solution, we propose creating a FHIR server, built upon an open-source FHIR implementation such as HAPI FHIR. The server would facilitate the interoperable exchange of medication data between existing hospital systems.

In a messaging-based approach, the server could act as a middleware between systems using FHIR messaging operations for real-time bidirectional communication, with clients publishing medication-related events that the server transforms and forwards. Either of these solutions will use the MII terminology service to validate medication codes. In the next stage of our project, we will validate this approach in our environment by developing an application for medication management.

Its primary objective is to address and solve local challenges while optimizing workflows for health care stakeholders both nationally and internationally in the context of health data management. By providing a nonproprietary, FAIR medication core dataset, our solution has the potential to be integrated into broader initiatives such as the European Health Data Space (EHDS) [55]. This integration would promote secure, efficient, and timely communication, as well as seamless use of electronic medication data across the EU.

Furthermore, it is designed to meet the needs of physicians, pharmacists, computer scientists, researchers, and administration personnel while adhering to national and international IT standards and guidelines, such as Germany’s Interop Council for Digital Health [56] and the WHO’s 2020 global strategy on digital health [57]. It aligns with the widely accepted perspective that standardization and interoperability are crucial for enhancing patient care and safety [58], improving workforce satisfaction and productivity, and fostering research and innovation. To the best of our knowledge, no comparable proposal has been presented in the literature.

### Limitations

This investigation has several limitations. Due to our institution’s large size, we did not consider medication data transfer and documentation of each general ward or ICU, potentially overlooking an already interoperable solution that could serve as a model for further development. However, given the widespread use of diverse medication software standards, an institution-wide analysis would likely reveal more interoperability conflicts when implementing any IT solution.

In addition, although HL7 version 2.3 is the most popular and widely used health care framework, we focused on FHIR due to its efficiency, flexibility, and suitability for mobile applications, her, and cloud communications [59,60]. Nonetheless, the database presented here could also be adapted for HL7 v.2.3 thanks to its standardized structure.

### Code Availability

All FHIR order messages were developed using Microsoft Visual Studio Code Version 1.90.2 and are available without restrictions on Figshare [61].

### Conclusions

Our results show that it is very challenging to achieve standardization and interoperability of medication records across the different IT platforms used in a hospital ecosystem.

To address this situation, we propose a solution that facilitates seamless data exchange among stakeholders using medication software, leveraging FHIR standards to overcome system disparities and data silos based on a noncommercial minimal, and FAIR medication dataset. This approach seeks to streamline digitization efforts across hospital departments, in key processes like patient discharge from the ICU to general wards. Beyond improving operational efficiency, our interoperable medication solution can help to reduce errors, enhance patient safety, and elevate the quality of care by enabling standardized data exchange and seamless stakeholder communication across the hospital ecosystem. Our study represents a conceptual proposal for a future solution at this stage, with no specific application or API interactions developed yet.

## Appendix

Multimedia Appendix 1 FHIR (Fast Healthcare Interoperability Resources) order message prototype with instructions for requesting a single medication (Propofol 1%) for a patient.

Multimedia Appendix 2 FHIR (Fast Healthcare Interoperability Resources) order message prototype with instructions for administration of a single medication (Propofol 1%) to a patient.

Multimedia Appendix 3 FHIR (Fast Healthcare Interoperability Resources) order message prototype with instructions for the request of a mixed medication (Noradrenaline and Sodium chloride [NaCl]) for a patient.

Multimedia Appendix 4 FHIR (Fast Healthcare Interoperability Resources) order message prototype with instructions for the administration of a mixed medication (Noradrenaline and Sodium chloride [NaCl]) to a patient.

## Abbreviations

ABDA: Federal Union of German Associations of Pharmacists
ABDA-MED: ABDA Medication Database
AI: artificial intelligence
API: application programming interface
ASK: German Drug Substance Catalog
ATC: Anatomical Therapeutic Chemical
BIH: Berlin Institute of Health
CLMA: closed-loop medication administration
CMIO: Chief Medical Informatics Officer
EDQM: European Directorate for the Quality of Medicines and HealthCare
EHDS: European Health Data Space
EHR: electronic health record
EMA: European Medicines Agency
FAIR: Findability, Accessibility, Interoperability, and Reusability
FHIR: Fast Healthcare Interoperability Resources
HL7: Health Level 7
ICD-10: *International Statistical Classification of Diseases, Tenth Revision*
ICU: intensive care unit
IDMP: Identification of Medical Products
ISO: International Organization for Standardization
LOINC: Logical Observation Identifiers Names and Codes
MedRec: Medication Reconciliation
MII: Medical Informatics Initiative
PDMS: patient data management system
PIS: pharmacy information system
PPN: Pharmacy Product Number
PZN: Pharmacy Central Number
SNOMED CT: Systematized Nomenclature of Medicine Clinical Terms
SPOR: Substance, Product, Organization, and Referential
TI: telematics infrastructure
UI: user interface
WHO: World Health Organization

## References

[1] (2020). Medical facilities, hospital beds and movement of patients. Destatis Statistisches Bundesamt.

[2] Biswal S, Mishra P, Malhotra S, Puri GD, Pandhi P (2006). Drug utilization pattern in the intensive care unit of a tertiary care hospital. J Clin Pharmacol.

[3] Valentin A, Capuzzo M, Guidet B, Moreno R, Metnitz B, Bauer P, Metnitz P, Research Group on Quality Improvement of the European Society of Intensive Care Medicine (ESICM), Sentinel Events Evaluation (SEE) Study Investigators (2009). Errors in administration of parenteral drugs in intensive care units: multinational prospective study. BMJ.

[4] Kohn LT, Corrigan JM, Donaldson MS, Institute of Medicine (US) Committee on Quality of Health Care in America (2000). To Err is Human: Building a Safer Health System.

[5] (2020). Sentinel event data summary. Joint Commission.

[6] Sanduende-Otero Y, Villalón-Coca J, Romero-García E, Díaz-Cambronero Ó, Barach P, Arnal-Velasco D (2020). Patterns in medication incidents: a 10-yr experience of a cross-national anaesthesia incident reporting system. Br J Anaesth.

[7] (2017). Medication without harm. World Health Organisation.

[8] Nelson SD, Parker J, Lario R, Winnenburg R, Erlbaum MS, Lincoln MJ, Bodenreider O (2017). Interoperability of medication classification systems: lessons learned mapping established pharmacologic classes (EPCs) to SNOMED CT. Stud Health Technol Inform.

[9] (2020). ECRI thought leadership. ECRI.

[10] Moges TA, Akalu TY, Sema FD (2022). Unintended medication discrepancies and associated factors upon patient admission to the internal medicine wards: identified through medication reconciliation. BMC Health Serv Res.

[11] Rahmawati HK, Adisasmito WBB (2019). The benefits of interoperability to prevent medication error in hospital.

[12] Agrawal A (2009). Medication errors: prevention using information technology systems. Br J Clin Pharmacol.

[13] Zeng ML (2019). Interoperability. Knowl Org.

[14] Benson T, Grieve G (2016). Snomed CT. Principles of Health Interoperability.

[15] Noumeir R (2019). Active learning of the HL7 medical standard. J Digit Imaging.

[16] (2021). About HL7. HL7 International.

[17] Ayaz M, Pasha MF, Alzahrani MY, Budiarto R, Stiawan D (2021). The fast health interoperability resources (FHIR) standard: systematic literature review of implementations, applications, challenges and opportunities. JMIR Med Inform.

[18] (2020). Revised IDMP standards to improve description of medicinal products worldwide. International Organization for Standardization (ISO).

[19] (2022). FDA Data Standards Advisory Board. FDA.

[20] (2020). Health informatics — identification of medicinal products — data elements and structures for the unique identification and exchange of regulated medicinal product information. International Organization for Standardization (ISO).

[21] (2020). Health informatics — identification of medicinal products — data elements and structures for unique identification and exchange of regulated pharmaceutical product information. International Organization for Standardization (ISO).

[22] (2020). Health informatics — identification of medicinal products — data elements and structures for the unique identification and exchange of regulated information on substances. International Organization for Standardization (ISO).

[23] (2020). Health informatics — identification of medicinal products — data elements and structures for the unique identification and exchange of regulated information on pharmaceutical dose forms, units of presentation, routes of administration and packaging. International Organization for Standardization (ISO).

[24] (2020). Data on medicines (ISO IDMP standards): overview. European Medicines Agency (EMA).

[25] (2020). Substance, product, organisation and referential (SPOR) master data. European Medicines Agency (EMA).

[26] (2020). European directorate for the quality of medicines and healthCare (EDQM) of the council of Europe. European Medicines Agency (EMA).

[27] (2020). Ph eur reference standards: orders and catalogue. European Directorate for the Quality of Medicines and HealthCare (EDQM).

[28] (2020). European pharmacopoeia - background and mission. European Directorate for the Quality of Medicines and HealthCare (EDQM).

[29] (2016). Data on medicines (ISO IDMP standards): overview. European Medicines Agency.

[30] Zabka S, Ammon D, Ganslandt T, Gewehr J, Haverkamp C, Kiefer S, Lautenbacher H, Löbe M, Thun S, Boeker M (2019). Towards a medication core data set for the medical informatics initiative (MII): initial mapping experience between the German procedure classification (OPS) and the identification of medicinal products (IDMP). Proceedings of the Joint Ontology Workshops.

[31] Drees D (2007). The Introduction of Health Telematics in Germany. ISSE/SECURE 2007 Securing Electronic Business Processes.

[32] (2022). Interoperability thanks to ISiK. gematik Fachportal.

[33] Wilkinson M, Dumontier M, Aalbersberg IJJ, Appleton G, Axton M, Baak A, Blomberg N, Boiten JW, da Silva Santos LB, Bourne PE, Bouwman J, Brookes AJ, Clark T, Crosas M, Dillo I, et al (2016). The FAIR guiding principles for scientific data management and stewardship. Sci Data.

[34] Queralt-Rosinach N, Kaliyaperumal R, Bernabé CH, Long Q, Joosten SA, van der Wijk HJ, Flikkenschild EL, Burger K, Jacobsen A, Mons B, Roos M, BEAT-COVID Group, COVID-19 LUMC Group (2022). Applying the FAIR principles to data in a hospital: challenges and opportunities in a pandemic. J Biomed Semantics.

[35] Barker M, Chue Hong NP, Katz DS, Lamprecht AL, Martinez-Ortiz C, Psomopoulos F, Harrow J, Castro LJ, Gruenpeter M, Martinez PA, Honeyman T (2022). Introducing the FAIR principles for research software. Sci Data.

[36] Du X, Dastmalchi F, Ye H, Garrett TJ, Diller MA, Liu M, Hogan WR, Brochhausen M, Lemas DJ (2023). Evaluating LC-HRMS metabolomics data processing software using FAIR principles for research software. Metabolomics.

[37] (2023). ABDAmed2: the complete package for hospitals and doctor's offices. ABDATA Pharma-Daten-Service.

[38] (2023). MMI pharmindex products. ViDAL mmi Germany.

[39] (2020). Expert knowledge for secure drug data. ABDATA Pharma-Daten-Service.

[40] Bouin AS, Wierer M (2014). Quality standards of the European pharmacopoeia. J Ethnopharmacol.

[41] (2020). Standard terms. European Directorate for the Quality of Medicines and HealthCare (EDQM).

[42] (2023). Resource medication. HL7 FHIR.

[43] Salgado-Baez E, Heidepriem R, Danhier RD, Rinaldi E, Poncette AS, Dahlhaus I, Fürstenau D, Balzer F (2024). FAIR IOP medication database. figshare.

[44] Lehne M, Sass J, Essenwanger A, Schepers J, Thun S (2019). Why digital medicine depends on interoperability. NPJ Digit Med.

[45] Bauer JC, John E, Wood C, Plass D, Richardson D (2020). Data entry automation improves cost, quality, performance, and job satisfaction in a hospital nursing unit. J Nurs Adm.

[46] Tripathi N, Goshisht MK, Sahu SK, Arora C (2021). Applications of artificial intelligence to drug design and discovery in the big data era: a comprehensive review. Mol Divers.

[47] Yang X, Wang Y, Byrne R, Schneider G, Yang S (2019). Concepts of artificial intelligence for computer-assisted drug discovery. Chem Rev.

[48] Liu C, Hogan AM, Sturm H, Khan MW, Islam MM, Rahman ASMZ, Davis R, Cardona ST, Hu P (2022). Deep learning-driven prediction of drug mechanism of action from large-scale chemical-genetic interaction profiles. J Cheminform.

[49] Austin JA, Smith IR, Tariq A (2018). The impact of closed-loop electronic medication management on time to first dose: a comparative study between paper and digital hospital environments. Int J Pharm Pract.

[50] Burkoski V, Yoon J, Solomon S, Hall TNT, Karas AB, Jarrett SR, Collins BE (2019). Closed-loop medication system: leveraging technology to elevate safety. Nurs Leadersh (Tor Ont).

[51] Ciapponi A, Fernandez Nievas SE, Seijo M, Rodríguez MB, Vietto V, García-Perdomo HA, Virgilio S, Fajreldines AV, Tost J, Rose CJ, Garcia-Elorrio E (2021). Reducing medication errors for adults in hospital settings. Cochrane Database Syst Rev.

[52] Parrish RH, Gilak L, Bohannon D, Emrick SP, Serumaga B, Guharoy R (2019). Minimizing medication errors from electronic prescription transmission-digitizing compounded drug preparations. Pharmacy (Basel).

[53] (2022). e-prescription: The fast way to the right medication. gematik.

[54] Salgado-Baez E, Näher AF, Friedrich M, Kremser G, Braune K, Balzer F, Henke V, Hülsken G, Schneider H, Varghese J (2024). Health data management im krankenhaus umsetzen. Health Data Management: Schlüsselfaktor für erfolgreiche Krankenhäuser.

[55] (2023). European health data space regulation (EHDS). European Comission.

[56] (2023). INTEROP COUNCIL for digital health in Germany.

[57] NA (2022). Global Strategy on Digital Health 2020-2025.

[58] Biltoft J, Finneman L (2018). Clinical and financial effects of smart pump-electronic medical record interoperability at a hospital in a regional health system. Am J Health Syst Pharm.

[59] (2020). Enabling health interoperability through FHIR. HL7 FHIR Foundation.

[60] Braunstein ML (2019). Health care in the age of interoperability part 6: the future of FHIR. IEEE Pulse.

[61] Salgado E, Heidepriem R, Danhier RD, Rinaldi E, Poncette AS, Dahlhaus I, Fürstenau D, Balzer F, Thun S, Sass J (2024). FHIR medication order messages prototypes. figshare.

