# Supplementary figures and images for: Toward Interoperable Digital Medication Records on Fast Healthcare Interoperability Resources: Development and Technical Validation of a Minimal Core Dataset
Source: JMIR Med Inform. 2025 May 9;13:e64099. doi: 10.2196/64099 (PMC12102619; doi:10.2196/64099)

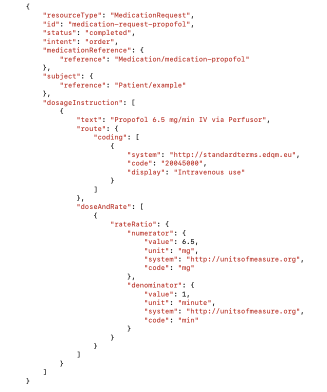

Supplement: Multimedia Appendix 1 [file medinform_v13i1e64099_app1.png]

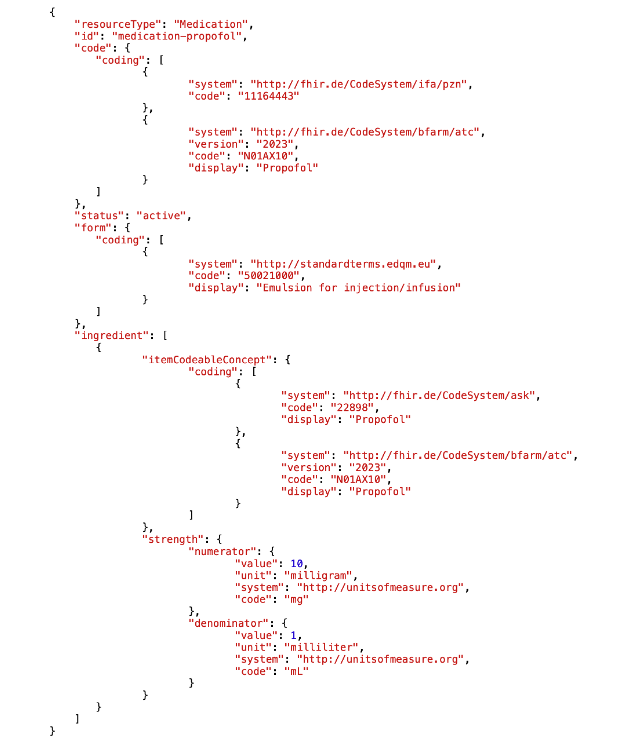

Supplement: Multimedia Appendix 2 [file medinform_v13i1e64099_app2.png]

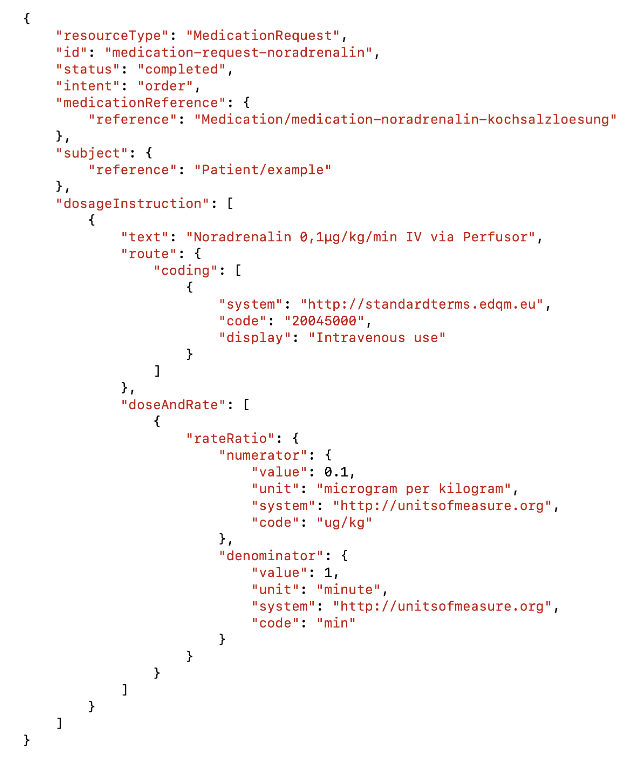

Supplement: Multimedia Appendix 3 [file medinform_v13i1e64099_app3.png]

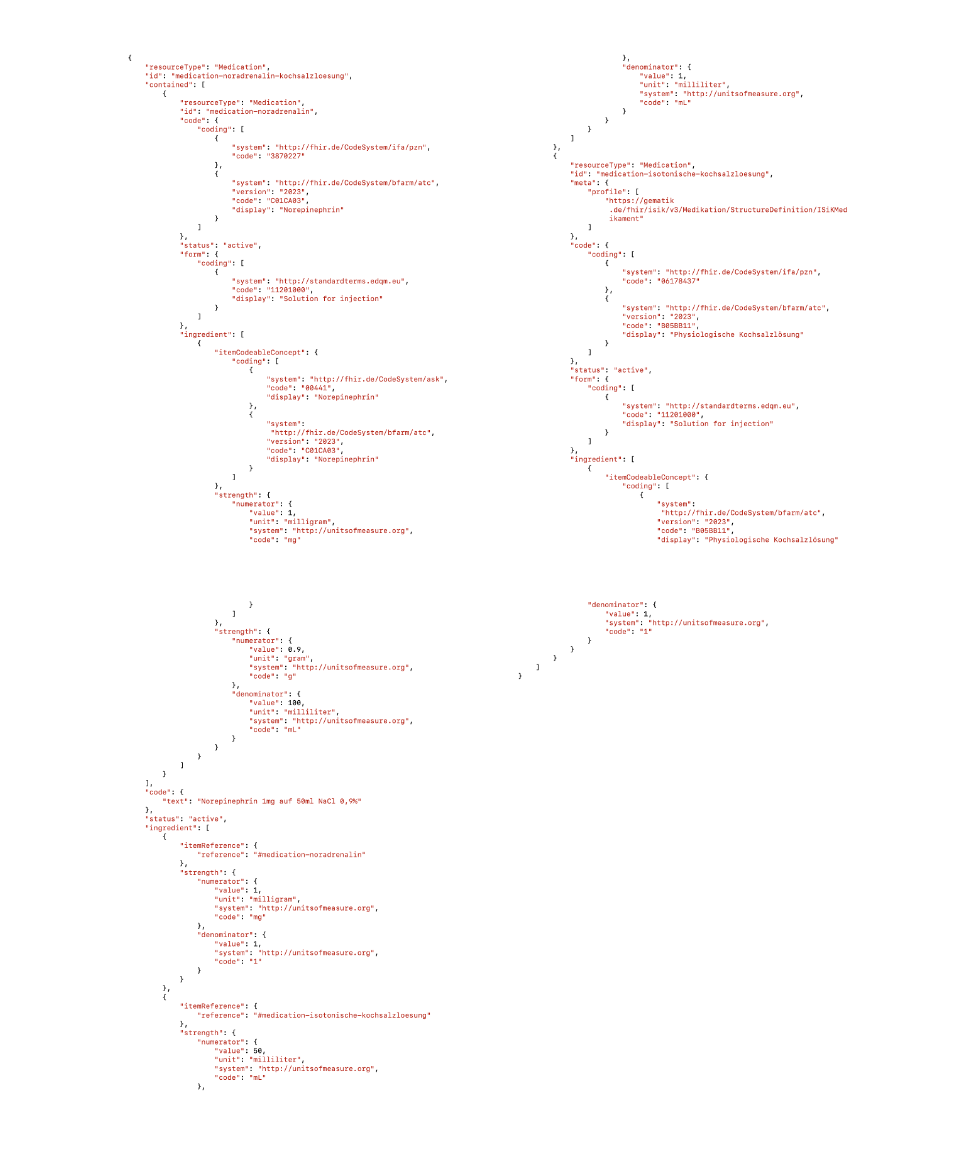

Supplement: Multimedia Appendix 4 [file medinform_v13i1e64099_app4.png]
